# Bio-hybrid scaffolds combining polyvinyl alcohol and decellularized articular cartilage for the treatment of focal chondral lesions in hemophilic patients

**DOI:** 10.3389/fphar.2025.1650467

**Published:** 2025-09-25

**Authors:** Silvia Barbon, Marta Confalonieri, Elena Stocco, Alice D’Osualdo, Martina Contran, Pier Paolo Parnigotto, Raffaele De Caro, Silvia Todros, Veronica Macchi, Piero G. Pavan, Andrea Porzionato

**Affiliations:** ^1^ Section of Human Anatomy, Department of Neuroscience, University of Padova, Padova, Italy; ^2^ Foundation for Biology and Regenerative Medicine, Tissue Engineering and Signaling – T.E.S. Onlus, Padova, Italy; ^3^ Department of Industrial Engineering, University of Padova, Padova, Italy; ^4^ Department of Women’s and Children’s Health, University of Padova, Padova, Italy; ^5^ Department of Surgery, Oncology and Gastroenterology, University of Padova, Padova, Italy

**Keywords:** polyvinyl alcohol, decellularization, articular cartilage, bio-hybrid scaffolds, mechanical behaviour, cartilage lesions, hemophilic arthropathy

## Abstract

**Introduction:**

Hemophilic arthropathy (HA) is a major complication of hemophilia, being caused by recurrent joint bleeding (hemarthrosis) which leads to iron accumulation in joints and subsequent damage to the articular cartilage (AC) and subchondral bone. Current treatments slow osteochondral degradation but do not promote cartilage regeneration. Tissue engineering offers a promising alternative for addressing AC damage in HA.

**Methods:**

This study developed bio-hybrid scaffolds based on polyvinyl alcohol (PVA) combined with decellularized human AC to enhance bioactivity and mechanical support. AC was harvested from cadaveric donors and decellularized via a detergent-enzymatic protocol, with efficacy confirmed through DNA quantification, histomorphometric analyses and Scanning Electron Microsopy (SEM). The resulting decellularized AC matrix was then homogenized in acetic acid for combination with PVA. Bio-hybrid scaffolds were produced using two fabrication methods: a) cross-linking of a freeze-dried AC layer onto the PVA hydrogel, forming a double-layer structure and b) mechanical incorporation of homogenized decellularized AC into the PVA matrix. Bio-hybrid scaffold morphology, porosity, and mechanical properties were analyzed, and cytocompatibility was evaluated by seeding HM1-SV40 human mesenchymal stem cells (MSC) and assessing cell adhesion and growth by MTT assays and SEM after 7 and 14 days.

**Results:**

Quality control studies on decellularized AC confirmed efficient cell/DNA removal and correct preservation of ECM components. Regarding the bio-hybrid scaffolds, SEM ultrastructural analysis revealed distinct surface roughness and porosity depending on the fabrication method. Compressive tests showed increased stiffness with higher PVA concentrations, while the addition of AC resulted in stiffness reduction, especially in the bilayer configuration. In parallel, consolidation tests revealed that PVA/AC_blend scaffolds showed similar short-term behavior to non-composite materials, while PVA/AC_bilayer exhibited a larger initial force drop, eventually aligning with PVA scaffolds at equilibrium. Cytocompatibility tests demonstrated that acellular AC matrix enhances PVA bioactivity, with PVA/AC_bilayer scaffolds better supporting cell adhesion and growth compared to PVA/AC_blend scaffolds.

**Discussion:**

The findings underscore the potential of PVA/AC bio-hybrid scaffolds for cartilage regeneration in hemophilic patients affected by HA. These scaffolds combine mechanical integrity with enhanced cytocompatibility, representing a novel strategy in the context of tissue-engineered therapies for joint repair.

## 1 Introduction

Hemophilia is a rare hereditary disease caused by a deficiency of specific blood clotting factors, which leads to the onset of haemorrhages, both internal, which occur spontaneously, and external, which occur following trauma or surgery. According to the levels of circulating clotting factor, the condition is defined to be mild (>5%), moderate (1%–5%) or severe (<1%) (Poenaru et al., 2023; Barbon et al., 2022a; Pulles et al., 2017; Bolton-Maggs and Pasi, 2003). In hemophilia patients, especially in case of severe condition, frequent joint bleeding (hemarthrosis) or muscle tissue bleeding (hematoma) can cause various complications. The most relevant is hemophilic arthropathy (HA), which is caused by repeated hemarthrosis at the target joints (especially the knee, but also the ankle and elbow joints), and represents the major reason of disability in hemophilia patients (Cuesta-Barriuso et al., 2022). Indeed, this repeated bleeding creates a severe inflammatory condition, damage to the cartilage, and, in the absence of adequate treatment, a progressive deterioration of the entire joint. Over time, this leads to pathological outcomes resembling osteoarthritis, such as pain, stiffness, limited mobility, and ultimately, joint deformities and functional impairment with a significant impact on patients’ quality of life (Gualtierotti et al., 2021). In particular, repeated joint bleeding induces synovitis, characterized by synovial hypertrophy, inflammation, and neoangiogenesis, which are driven by iron deposition and oxidative stress. Pro-inflammatory mediators (IL-1β, TNF-α, ROS, MMPs) promote chondrocyte apoptosis and impair cartilage matrix synthesis (Melchiorre et al., 2017). Articular cartilage damage, caused by both inflammatory and mechanical processes, further reduces chondrocyte viability and extracellular matrix (ECM) integrity.

At present, hemophilic arthropathy (HA) is managed through a multidisciplinary approach combining pharmacological therapy and clinical-surgical interventions (Carulli et al., 2020). The pharmacological management is mainly based on coagulation factor replacement therapy, administered either “on demand” during bleeding episodes or as regular prophylaxis to prevent them. Prophylactic infusions, ideally initiated early in life before the onset of joint damage, have significantly reduced bleed frequency and delayed HA progression (Manco-Johnson et al., 2007). However, prophylaxis is a costly approach that requires strict lifelong intravenous administration, which can negatively impact venous access and treatment adherence (Carcao and Manco-Johnson, 2021) and may lead to the development of neutralizing antibodies (inhibitors) that reduce treatment efficacy (Meeks et al., 2019).

More recently, non-factor therapies such as emicizumab (for hemophilia A) have emerged, offering subcutaneous administration and effective bleed reduction (Oldenburg et al., 2017; Pipe et al., 2020). Nevertheless, these treatments are not curative, require indefinite use, and have limited long-term safety data (Shima et al., 2021).

When HA progresses despite optimal medical management, conservative orthopedic strategies are employed. These include physiotherapy to preserve mobility (Gao et al., 2023), intra-articular corticosteroid or hyaluronic acid injections to control pain and inflammation (Buccheri et al., 2019; Rodríguez-Merchán, 2023), and orthotic devices to reduce joint stress (Wilkins et al., 2022). While these measures can alleviate symptoms and slow disease progression, they do not reverse cartilage damage and may require repeated interventions, which carry risks such as infection or post-procedural bleeding.

Surgical options, such as synovectomy (arthroscopic, chemical, or radioisotopic), osteotomy, or joint arthroplasty, are reserved for advanced disease (Rodríguez-Merchán, 2019). While they can improve function and quality of life, these procedures are associated with elevated perioperative bleeding risk in hemophilia patients, and, in the case of arthroplasty, limited prosthesis longevity in younger individuals (Konkle et al., 2019).

Overall, current HA treatments aim to minimise bleeding and decrease pain, only succeeding in slowing down the progression of joint damage (Gualtierotti et al., 2021).

In this context, tissue engineering (TE) strategies have been proposed as a possible therapeutic solution to be adopted in the earliest stages of HA onset and development, with the aim to regenerate focal AC damages caused by the joint bleeding. According to this approach, biomaterial-based scaffolds can be used as supportive three-dimensional (3D) frameworks that promote cell growth/differentiation and stimulate AC regeneration, effectively restoring the joint. By targeting pathological degenerative changes in the early stages of HA, this strategy could potentially halt or delay the progression of the disease, reducing the need for joint replacement and mitigating the personal and socio-economic burden associated with HA (Liang et al., 2023; Tamaddon et al., 2020; Stocco et al., 2016).

Although natural and synthetic polymeric materials have been widely investigated in cartilage tissue engineering, their application in HA remains limited. To date, polymer-based strategies have focused predominantly on intra-articular viscosupplementation with hyaluronic acid hydrogels, which represent the most extensively studied systems in HA (Arnold et al., 2000; Melchiorre et al., 2002; Melchiorre et al., 2012; Rodriguez-Merchan, 2013; Solimeno et al., 2019; Melchiorre et al., 2022). More recently, innovative preclinical approaches have begun to explore alternative polymers. Yang and colleagues (2024) developed a catechol-functionalized gelatin hydrogel with strong tissue adhesion, used to deliver mesenchymal stem cell-derived exosomes and promote cartilage repair in hemophilic joints. Zhang et al. (2025) introduced a synthetic multicomponent hydrogel with polyphenol, chelating, and lipoic groups, forming an injectable, self-healing, and antioxidant matrix for intra-articular therapy. These studies illustrate how both natural and synthetic polymer strategies can be tailored to address the complex joint environment in HA, extending beyond traditional hyaluronic acid viscosupplementation.

Among the most promising biomaterial candidates to treat HA, polyvinyl alcohol (PVA) hydrogels have been widely studied as synthetic replacements for AC (Wei and Dai, 2021; Grant et al., 2006), due to their excellent ductility, flexibility and mechanical strength. Specifically, PVA viscoelastic behavior, compressive modulus, shear modulus, tensile modulus, and permeability adequately resemble those of natural cartilage, making the polymer highly appealing for cartilage TE applications (De Leon-Oliva et al., 2023; Spiller et al., 2011). In addition, PVA is characterized by low toxicity and adjustable porosity, which can be tailored through the number of freezing-thawing (FT) cycles used during scaffold polymerization (Todros et al., 2022; Barbon et al., 2021; Baker et al., 2012). Commercial PVA-based devices, such as SaluCartilage™ (Salumedica, Smyrna, GA), Cartiva™, and Cartiva SCI™ (Carticept Medical, Inc.) are specifically designed to replicate the properties of natural AC, being currently used in the clinical setting to repair focal cartilage defects while minimizing the need for healthy tissue resection. However, despite polymer biocompatibility, PVA has little ability to promote cell adhesion (Stocco et al., 2014). To overcome this limitation, several strategies have been investigated to increase PVA bio-activity, including the combination of the polymer with natural/biological materials. Among these materials, decellularized ECM derived from human AC offers the great advantage of maintaining the native environment of the tissue to be repaired, thus enhancing the adhesion and growth of chondrocytes that will guide cartilage regeneration. Indeed, the decellularization process is crucial for removing immunogenic components of the native tissue, such as cells and nucleic acids, while retaining bio-active elements of the original ECM, including growth factors, polysaccharides, and structural proteins. These components create a favorable environment for cell proliferation and effectively stimulate the regenerative process (Stocco et al., 2023; Barbon et al., 2022b; Barbon et al., 2022c).

Starting from the above considerations, this study aimed to fabricate and compare bio-hybrid scaffolds composed of PVA - providing mechanical support - and decellularized human AC -enhancing biological function - for potential application in the early-stage treatment of HA.

For scaffold fabrication, two polymer concentrations (15% and 20% PVA) were selected to evaluate mechanical properties in relation to native cartilage. In parallel, decellularized AC was integrated into the PVA matrix using two distinct methods: 1) *bilayer configuration*, in which an acellular AC layer was placed on top of the polymer, mimicking the zonal structure of cartilage and allowing direct interaction of cells with the tissue-like surface (i.e., 15%PVA/AC_bilayer and 20%PVA/AC_bilayer scaffolds); and 2) *blend configuration*, where acellular AC was homogenously dispersed within the polymer, promoting a more uniform distribution of biological cues throughout the scaffold (i.e., 15%PVA/AC_blend and 20%PVA/AC_blend).

Combining bioactive cartilage ECM with PVA hydrogel would improve the biological performance of the synthetic material, and that the fabrication strategy - bilayer versus blend - could significantly influence scaffold architecture, mechanical integrity, and cell compatibility.

The rationale for comparing these two fabrication approaches lies in their complementary advantages: the bilayer strategy tries to replicate the spatial organization of native AC with a distinct surface zone, while the blend configuration is designed to create a uniformly bioactive and mechanically integrated structure.

The resulting scaffolds were then analysed for their morphological, structural, mechanical, and cytocompatibility properties to evaluate their potential use in regenerative approaches addressing haemophilic cartilage degeneration.

## 2 Materials and methods

### 2.1 Reagents and materials

Reagents and materials used for this research were mainly purchased from the Company Merck Life Science (Darmstadt, Germany), unless otherwise specified.

### 2.2 Decellularization of human articular cartilage (AC)

Human cartilage samples for AC matrix fabrication (n = 3, age range: 69–73 years old) were harvested from cadaver donors participating in the Body Donation Program of the Section of Human Anatomy, University of Padova (De Caro et al., 2009) in adherence with European, Italian, and Regional regulations (Boscolo-Berto et al., 2020). Following collection, the tissue samples underwent multiple rinses in a 2% penicillin/streptomycin solution in phosphate-buffered saline (PBS) to eliminate blood residuals. Thus, the cartilage was fragmented into smaller pieces and subjected to the decellularization process using a detergent-enzymatic approach as described by Stocco and Collaborators (Stocco et al., 2014). Specifically, samples were kept immersed under stirring in distilled water (dH_2_O) for 72 h (h) at +4 °C, replacing the aqueous solution every 2 h. Subsequently, the tissue was first treated with 4% sodium deoxycholate (≥97% (titration), Sigma Aldrich, St. Louis, MI, United States) for 4 h at room temperature (RT), and then with 2,000 KU (Kunitz Units) of Deoxyribonuclease I (DNase-I from bovine pancreas) in 1 M sodium chloride (NaCl) solution for 2 h at RT. The decellularization was repeated for three cycles. To prevent any contamination, the tissue was always manipulated under sterile conditions, including the use of a laminar flow hood, sterile instruments and aseptic decellularization solutions.

The yield of decellularized AC obtained from each donor was sufficient for all subsequent experimental analyses. No major technical difficulties were encountered, and the decellularization process did not cause detectable cartilage loss, with the initial sample mass maintained across each cycle, ensuring reproducibility and reliability of the procedure.

### 2.3 Characterization of the decellularized AC matrix

#### 2.3.1 Nuclear staining with 4′,6-Diamidino-2-Phenylindole (DAPI)

To assess the effective removal of cell and DNA components after the decellularization, the nuclear staining with DAPI fluorescent dye was performed. Both native and acellular AC samples were embedded in optimal cutting temperature (OCT) compound (VWR Chemicals, Radnor, PA, United States), frozen at −80 °C for at least 24 h and cut into 5 µm-thick sections using a cryomicrotome (Leica CM1850 UV) (Leica, Wetzlar, Germany). After air drying, the sections were immersed in dH_2_O for 15 min (minutes) prior to be incubated in darkness with DAPI solution for 10 min. Post-incubation, the sections were washed with PBS and then mounted with Mowiol^®^ mounting medium. Photomicrographs were then captured using microscopy equipment consisting of a Leica LMD6 microscope, a Leica DFC320 high-resolution digital camera, and an associated image acquisition software (LasX, Leica).

#### 2.3.2 Residual DNA quantification

To evaluate the efficiency of the decellularization protocol, DNA content was quantified in acellular and native AC tissues using the DNeasy Blood and Tissue Kit (Qiagen, Düsseldorf, Germany). In brief, 12 mg of tissue were lysed by Proteinase K at +56 °C, overnight. The resulting lysates underwent DNA purification using DNeasy Mini spin columns to achieve selective isolation of DNA. DNA quantification was then performed by a fluorometric method using a Qubit 4 fluorometer and corresponding kit (ThermoFisher Scientific, Waltham, MA, United States). Three replicates for each experimental group were considered.

#### 2.3.3 Histological analyses

The correct preservation of morpho-structural features and ECM components after decellularization was assessed by means of histological analyses conducted on acellular versus native AC samples. Specifically, Hematoxylin and Eosin (H&E) staining assessed the removal of the cellular component and the preservation of the original AC histoarchitecture; Masson’s Trichrome and Picrosirius Red stainings verified the preservation of the collagen fibers and Alcian Blue staining was performed to demonstrate the permanence of the glycosaminoglycan (GAG) component. For these analyses, AC samples were fixed in 10% (v/v) formalin for at least 72 h and processed with a tissue processor (Leica TP 1020). After embedding in paraffin, the samples were cut in 5 µm-thick sections using a microtome (Leica RM2135). Thus, the sections were stained in accordance with previously described protocols which are routinely used in our laboratory (Stocco et al., 2023; Barbon et al., 2022b; Barbon et al., 2022c).

#### 2.3.4 Morphometric analyses

Semi-quantitative morphometric analysis of collagen components in decellularized and native AC sections was conducted using Masson’s Trichrome- and Picrosirius Red-stained sections. Morphometric studies were performed by using ImageJ software (version 1.53c, U.S. National Institutes of Health, Bethesda, MD, United States) and image analysis procedures previously reported in the literature (Stocco et al., 2023; Barbon et al., 2022c). The collagen content was quantified as the percentage of areas stained in green (collagen) and red (type I collagen) in Masson’s Trichrome, and Picrosiurius Red stains, respectively. Images of stained sections were captured from eight distinct fields per section for each experimental group, using bright-field microscopy or polarized light microscopy in case Picrosiurius Red staining, at ×20 magnification. After saving images as TIFF files, green and red areas were identified by analyzing histograms of hue, saturation, and brightness distributions, and applying specific thresholds: 66–113 (hue), 0–255 (saturation), and 219–253 (brightness) for green (collagen); 0–42 (hue), 0–255 (saturation), and 0–255 (brightness) for red (type I collagen). The corresponding color ranges were manually selected and consistently applied across all analyses. Results are expressed as the percentage of green or red areas relative to the total area of the acquisition field.

#### 2.3.5 GAG quantification

Sulphated GAGs in decellularized AC samples and native tissue were quantified using the Chondrex Inc. Glycosaminoglycans Assay Kit (DBA Italia S.r.l., Milan, Italy). Tissue samples weighing 10 mg were digested overnight at +56 °C in a Papain solution to solubilize GAGs, which were subsequently labeled with the cationic dye 1,9-dimethylmethylene blue (DMB). The resulting colorimetric reaction was measured at 530 nm using the VICTOR3™ Microplate Auto Reader (PerkinElmer, Waltham, MA, United States). Chondroitin sulfate was included as a standard for GAG quantification in the samples.

#### 2.3.6 Ultrastructural analysis by scanning electron microscopy (SEM)

Native and decellularized AC ultrastructure was observed by Scanning Electron Microscopy (SEM). Sample preparation for SEM analysis involved a series of sequential steps: fixation in 2.5% glutaraldehyde in 0.2 M phosphate buffer (pH 7.4) for at least 24 h, dehydration through washes in ethanol at increasing concentrations (30%, 40%, 50%, 60%, 70%, 80%, 90% for 45 min/each; 95% overnight; 100% for 30 min) for 45 min each, critical point drying and gold coating using the gold sputtering technique. The prepared samples were then observed using a Scanning Electron Microscope (JSM-6490LA, JEOL, Eching b. München, Germany).

### 2.4 Preparation of the homogenized acellular AC matrix

The decellularized cartilage fragments (1 g) were immersed in 15 mL of 10% acetic acid solution (2.5 M) in dH_2_O to extract the protein fraction. Then, keeping the sample in an ice bath at approximately +4 °C to prevent overheating, the cartilage was homogenized using an Ultra-Turrax homogenizer (Janke & Kunkel GmbH, Staufen, Germany). The homogenization process involved eight cycles of 20 s (seconds) each, with 5 min-intervals between cycles.

Once the homogenization was completed, one part of the destructured matrix was stored at −20 °C in a 50 mL tube, for the fabrication of the composite blend scaffolds. The second part was aliquoted into a 48-well plate (300 µL/well), placed at −20 °C overnight and freeze-dried with a freeze-dry system (FreeZone, Labconco Corporation, Kansas City, United States) to obtain matrix sheets to be placed on PVA for bilayer scaffold fabrication.

### 2.5 Preparation of PVA solutions and hydrogels

Aqueous solutions of PVA (Mw 146,000–186,000 Da, 99+% hydrolysed) at 15% and 20% (w/v) were obtained by dissolving the corresponding amount of polymer powder in dH_2_O. Polymer concentrations of 15% and 20% were selected based on previous reports indicating that this range ensures optimal viscosity for scaffold fabrication and mechanical properties resembling those of articular cartilage (Chen et al., 2021; Adelnia et al., 2022).

The PVA suspensions were heated for 8 h at +90 °C under stirring until complete dissolution of the powder. The respective PVA samples will be denoted as 15%PVA and 20%PVA, based on their polymer concentration.

To prepare the hydrogel scaffolds, PVA solutions were poured between two glass plates separated by 2 mm-thick spacers and underwent physical cross-linking using a previously standardized method (Barbon et al., 2020). Specifically, PVA aqueous solution was frozen at −20 °C for 24 h and following thawed at +4 °C for other 24 h. This process was repeated for 3 FT cycles, after which the hydrogels were stored at −20 °C until further use.

### 2.6 Fabrication of the bio-hybrid PVA/AC scaffolds

#### 2.6.1 Fabrication of the bilayer scaffold

The PVA/AC bilayer scaffold consists of two layers: a lower layer made of PVA and an upper layer made of acellular cartilage matrix. To prepare this scaffold, the PVA solution (15% or 20%) was heated to approximately +60 °C and poured into a well of a 48-well plate to form a 2-mm thick polymer layer. After that, a sheet of homogenized and freeze-dried acellular cartilage matrix was gently placed on the surface of the polymer. Finally, PVA was physically cross-linked through 3 FT cycles (F: 24 h at −20 °C, T: 24 h at +4 °C). The cross-linking not only served for hydrogel polymerization, but also facilitated adhesion between the AC matrix and the polymer.

#### 2.6.2 Fabrication of the blend scaffold

The PVA/AC blend scaffold is a single-layer support where the acellular cartilage matrix is mixed with PVA. The scaffold was fabricated by mechanically incorporating the homogenized acellular AC into the polymer. Specifically, 400 µL of decellularized matrix were incorporated into 2 g of 15% or 20% PVA solution, pre-heated at +60 °C, using a stainless-steel laboratory spatula; subsequently, the composite was cast between two glass plates separated by a 2 mm spacer. Polymer cross-linking was achieved through 3 FT cycles (F: 24 h at −20 °C, T: 24 h at +4 °C). At the end of the cross-linking process, 7 mm-diameter discs were obtained using a biopsy punch.

#### 2.6.3 Ultrastructural analysis and porosity measurements

After fabrication of the PVA/AC_bilayer and PVA/AC_blend scaffolds, their superficial ultrastructure was investigated by SEM. The bio-hybrid scaffolds were fixed, processed and observed as already described in paragraph 2.3.6.

Scaffold superficial porosity was measured on SEM images at ×5000 magnification by using the ImageJ software. Briefly, 8-bit image type was selected the threshold tool was used to select the areas of porosity. Specifically, the threshold values were adjusted to select the dark porosity spots which became red. After applying the threshold parameter to obtain a binary image of the porosity, the “Analyze particles” function was used to measure the area fraction value (i.e., percentage of porosity) and the average size of the selected porosity spots.

### 2.7 Mechanical tests on bio-hybrid PVA/AC scaffolds

Compressive tests were carried out on the Bose ElectroForce^®^ Planar Biaxial Test Bench instrument (TA Instruments, New Castle, DE, United States). Five cylindrical samples (diameter: 7 mm, thickness: 2 mm) for each experimental group were tested, specifically 15%PVA, 15%PVA/AC_bilayer and 15%PVA/AC_blend, 20%PVA, 20%PVA/AC_bilayer and 20%PVA/AC_blend. The samples were let thaw in PBS at RT for 1 h before testing. The hydration condition was maintained for the whole duration of the test. The contact of the samples with a flat compressive surface was defined when recording a compressive force of −0.005 N. Then, the samples were compressed with a strain rate of 150% s^−1^, till reaching the 20% of the initial height of compression. Consolidation was achieved by keeping the maximum deformation for 3,600 s. Nominal stress and nominal strain were calculated dividing the compressive force by the initial superficial area and dividing the displacement by the undeformed sample thickness. The mean nominal stress and the standard error were determined for each experimental group. The compressive stiffness was calculated as the secant modulus of the nominal stress–nominal strain curve at 20% strain. Moreover, the normalized force was calculated as the ratio between the current force and the maximum compressive force. The mean values of normalized force were calculated for each experimental group.

### 2.8 Cytocompatibility test

#### 2.8.1 HM1-SV40 cell culture

For seeding experiments on the bio-hybrid PVA/AC scaffolds, the immortalized Human Bone Marrow Mesenchymal Stromal Cell (MSC) line HM1-SV40 was used. This cell population was cultured in T25 cell culture flasks (Corning, NY, United States) at +37 °C, 95% relative humidity and 5% CO_2_, using a proliferation medium containing Alfa-Modified Eagle Medium (α-MEM) Without Nucleosides (ThermoFisher Scientific, Waltham, MA, United States), 16.5% fetal bovine serum (FBS) (ThermoFisher Scientific), 1% L-Glutamine 200 mM and 1% penicillin/streptomycin solution (100 mg/mL) (P/S; Life Technologies, Paisley, United Kingdom). The medium was refreshed every 2–3 days.

#### 2.8.2 Cell seeding on bio-hybrid scaffolds

Before cell seeding, the bio-hybrid PVA/AC scaffolds underwent a sterilization process including several steps. The scaffolds were initially immersed in an antibiotic solution consisting of 2% penicillin/streptomycin in PBS for 24 h under agitation, with the solution replaced every 2 h. Subsequently, the scaffolds were placed in PBS to remove any antibiotic residues, changing the buffer every 2 h. Finally, the samples were irradiated with UV light for 40 min and incubated at +37 °C overnight into HM1-SV40 proliferation medium.

To perform cell seeding on scaffolds, the cells were detached from the culture flask using a trypsin solution and counted with Trypan Blue dye and an automated cell counter (TC20 Automated Cell Counter, BIO-RAD, Hercules, CA, United States). After removing the culture medium used to prepare the PVA/AC supports, 100,000 cells per scaffold were seeded by gently adding 50 µL of cell suspension onto the scaffold surface. Cells were allowed to adhere on the bio-hybrid supports in very low medium volume for 15 min at +37 °C, 95% humidity, and 5% CO_2_. After that, culture medium was added to completely cover the scaffolds.

#### 2.8.3 Verification of cell growth on scaffolds by MTT assay

The 3-(4,5-dimethylthiazol-2-yl)-2,5-diphenyltetrazolium bromide (MTT) was used to assess HM1-SV40 cell viability and proliferation on bio-hybrid PVA/AC scaffolds at 7 and 14 days from seeding. After removing the proliferative medium, the samples were treated with a 10% solution of MTT (5 mg/mL) in α-MEM basal medium, for 4 h at +37 °C. At the end of the incubation, the formazane crystals were collected and solubilised in 2-propanol acid (HCl 0.04 M in 2-propanol) (Carlo Erba, Milan, Italy) for 15 min under agitation. The absorbance of the resulting solution, directly proportional to the number of viable cells on each scaffold, was measured at a wavelength of 570 nm with the VICTOR3™ automated microplate reader (PerkinElmer, Waltham, MA, United States).

#### 2.8.4 Ultrastructural analysis by SEM

The ultrastructural morphology of HM1-SV40 cells which adhered and grew on the PVA/AC scaffolds, both bilayer and blend, was assessed by SEM imaging. The seeded samples were fixed, processed and observed as already described in paragraph 2.3.6.

### 2.9 Statistical analysis

Results are presented as the mean ± standard deviation of at least 3 replicates for each experiment. Statistical analysis was conducted using Prism software (version 9.3.1, GraphPad Software, San Diego, United States). Differences between experimental groups were analysed using the non-parametric Mann-Whitney test for comparisons between two groups, and One‐way ANOVA followed by Dunnett’s post hoc test for multiple group comparisons. Statistical significance was set at p ≤ 0.05.

## 3 Results

### 3.1 Decellularization effectively removes immunogenic components while preserving ECM structure and composition

The effectiveness of the decellularization process, performed by a detergent-enzymatic treatment, was verified through DAPI nuclear labeling, H&E staining and residual DNA quantification. By comparing native AC fragments with treated samples, these analyses enabled the assessment of both the effective removal of immunogenic components (i.e., DNA and cells) and the preservation of the basic AC structure. By comparing native AC fragments with treated samples, these analyses enabled the assessment of both the effective removal of immunogenic components of the tissue (i.e., DNA and cells) and the preservation of the basic AC structure.

**FIGURE 1:**
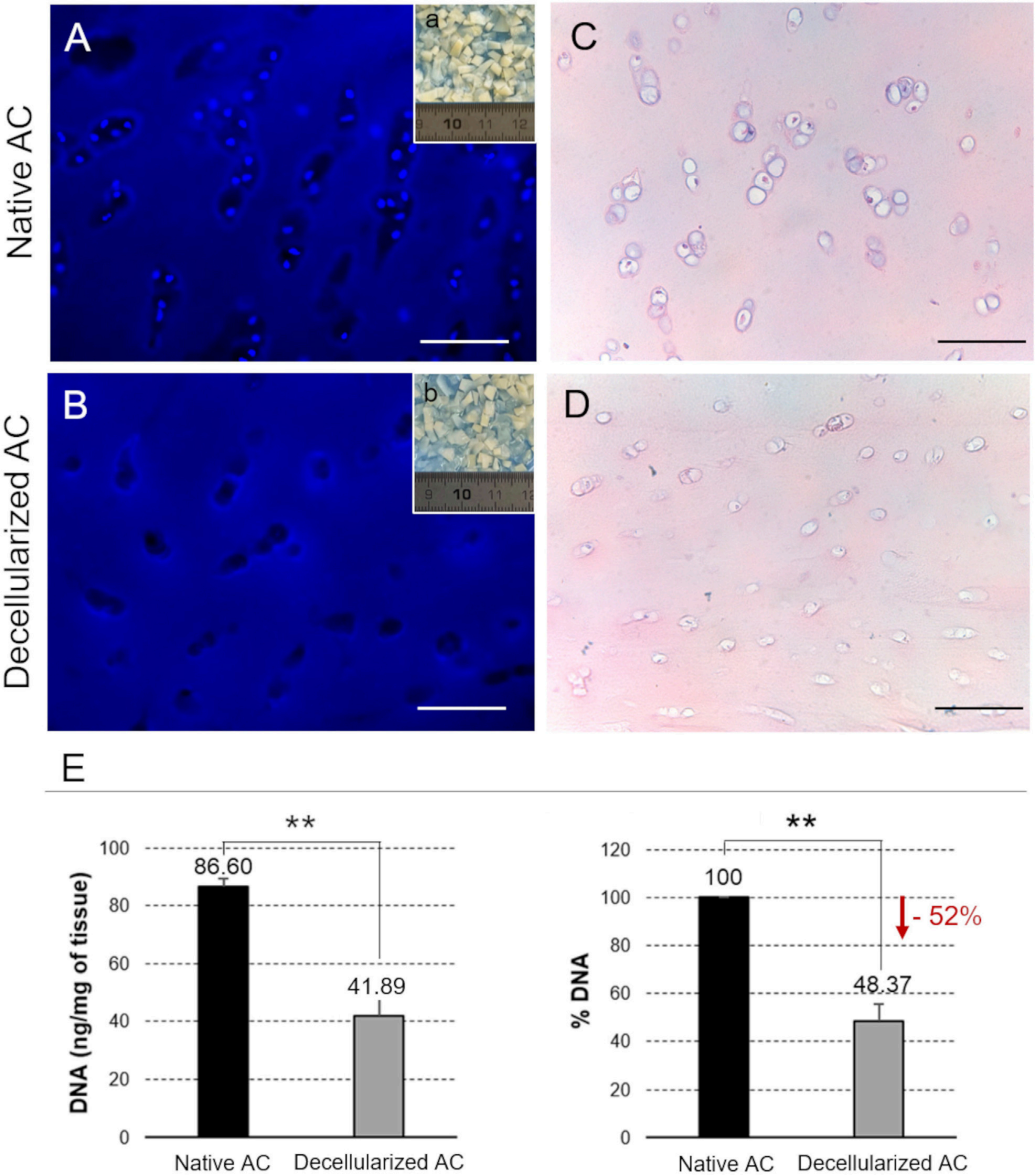
Depletion of the immunogenic components. **(a,b)** Gross appearance of the AC fragments before (Native AC) and after the decellularization treatment (Decellularized AC). **(A,B)** Nuclear labelling with DAPI of native versus decellularized AC. **(C,D)** Histological analysis by H&E staining of the cell components and microscopic structure of native versus decellularized AC samples. Scale bar: 100 µm. **(E)** Quantification of residual DNA into the AC fragments before and after the detergent-enzymatic treatment (**p < 0.01).

Macroscopically, the decellularized AC fragments derived from donated human cartilage showed a certain whitening and became more translucent in comparison with the native tissue (Figures 1a,b). DAPI staining confirmed the effective depletion of cell nuclei following decellularization (Figures 1A,B). H&E staining corroborated efficient cell removal and indicated no alteration in the microscopic AC structure after the detergent-enzymatic treatment, with typical cartilage lacunae being well identifiable and containing chondrocytes in the native but not in the decellularized samples (Figures 1C,D). The residual DNA content in the decellularized AC was measured at 41.89 ± 6.43 ng/mg, which is below the 50 ng/mg threshold commonly reported in the literature for non-immunogenic acellular matrices (Crapo et al., 2011). The decellularization treatment removed about the 52% of the initial genetic material (Figure 1E).

**FIGURE 2:**
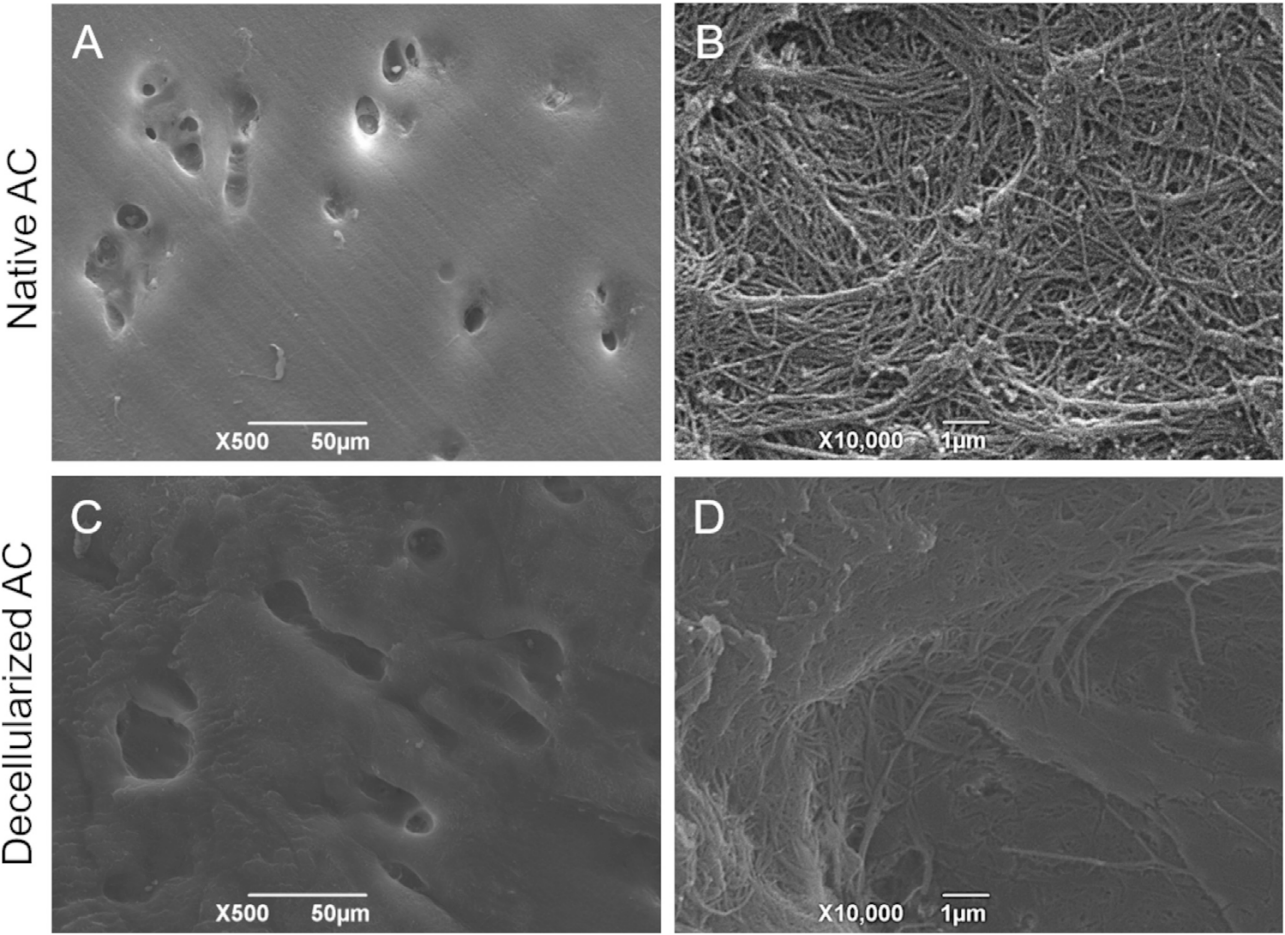
Ultrastructural analysis on AC tissue. SEM micrographs showing the typical ultrastructure of native **(A,B)** *versus* decellularized **(C,D)** AC fragments. Scale bar: 50 µm **(A,C)**; 1 µm **(B,D)**.

Scanning Electron Microscopy investigation revealed that the ultrastructure of AC tissue was maintained after the decellularization process. Cartilage lacunae can be appreciated in lower magnification micrographs (Figures 2A,C), whereas the abundant collagen component organized into a dense network of fibers is well recognizable at higher magnification (Figures 2B,D).

**FIGURE 3:**
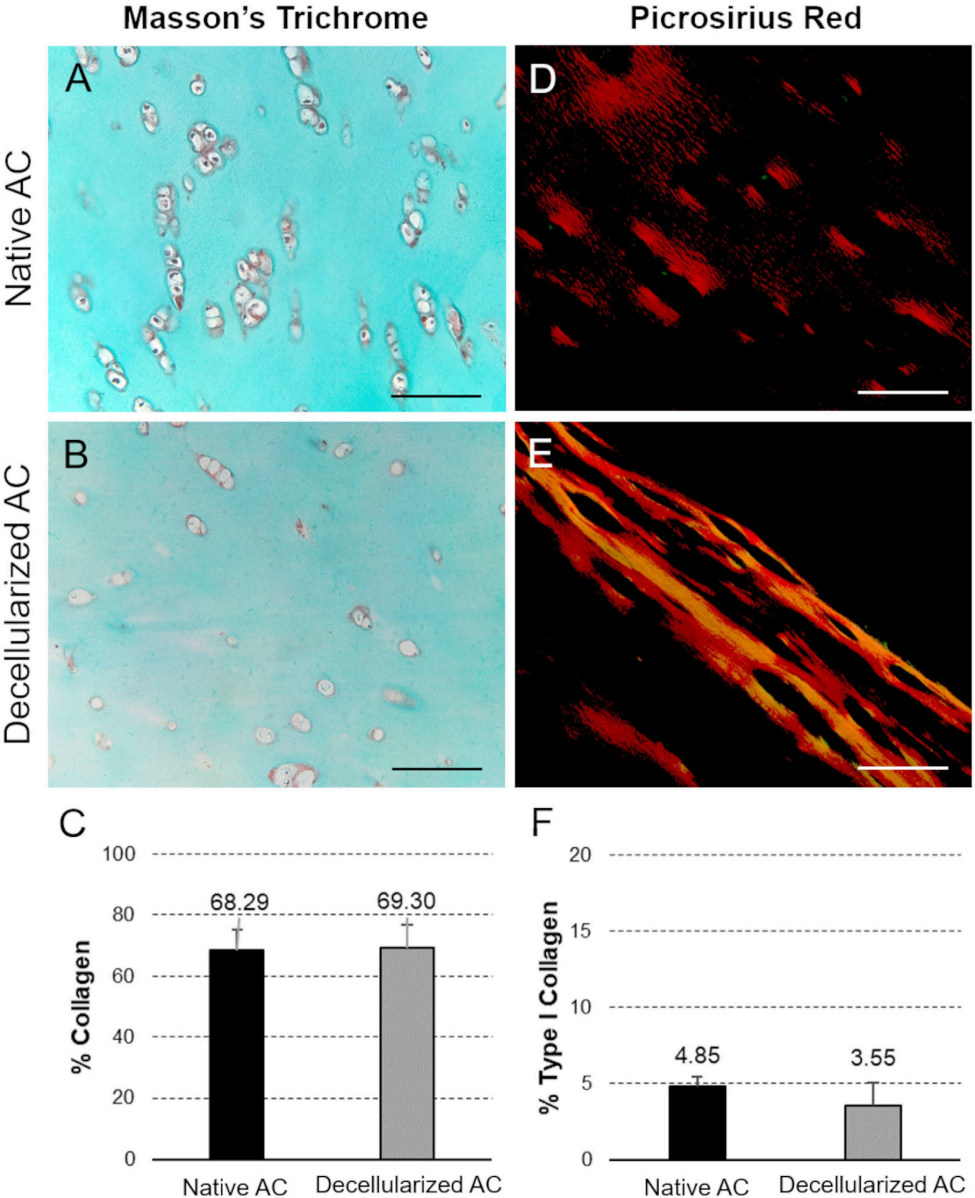
Collagen fiber preservation. Masson’s Trichrome **(A,B)** and Picrosirius Red **(D,E)** staining of the collagen component into decellularized versus native AC fragments. Scale bar: 100 µm. Morphometric analysis of histological sections provided semi-quantitative data on collagen expression in AC samples before and after the detergent-enzymatic treatment **(C,F)**.

Besides removing the immunogenic cues, the second main goal of decellularization is to correctly preserve the original ECM composition, since biomolecules like collagen and GAGs play a crucial role in providing structural support to tissues and serve as key regulators of cell-matrix interactions. Based on this, collagen preservation in decellularized versus native AC fragments was assessed using Masson’s Trichrome (total collagen; Figures 3A,B) and Picrosirius Red (type I collagen; Figures 3D,E) staining. Both samples showed positive staining, with green color of the ECM indicating total collagen and red color indicating type I collagen, respectively. Moreover, Masson’s Trichrome stain also confirmed the absence of cell nuclei at the chondral lacunae, which, in contrast, can be identified as black-colored elements into the native tissue (Figures 3A,B).

Morphometric analyses on histological sections revealed no significant differences among the 2 experimental groups, confirming the correct preservation of ECM collagen fibers after decellularization (Figures 3C,F).

**FIGURE 4:**
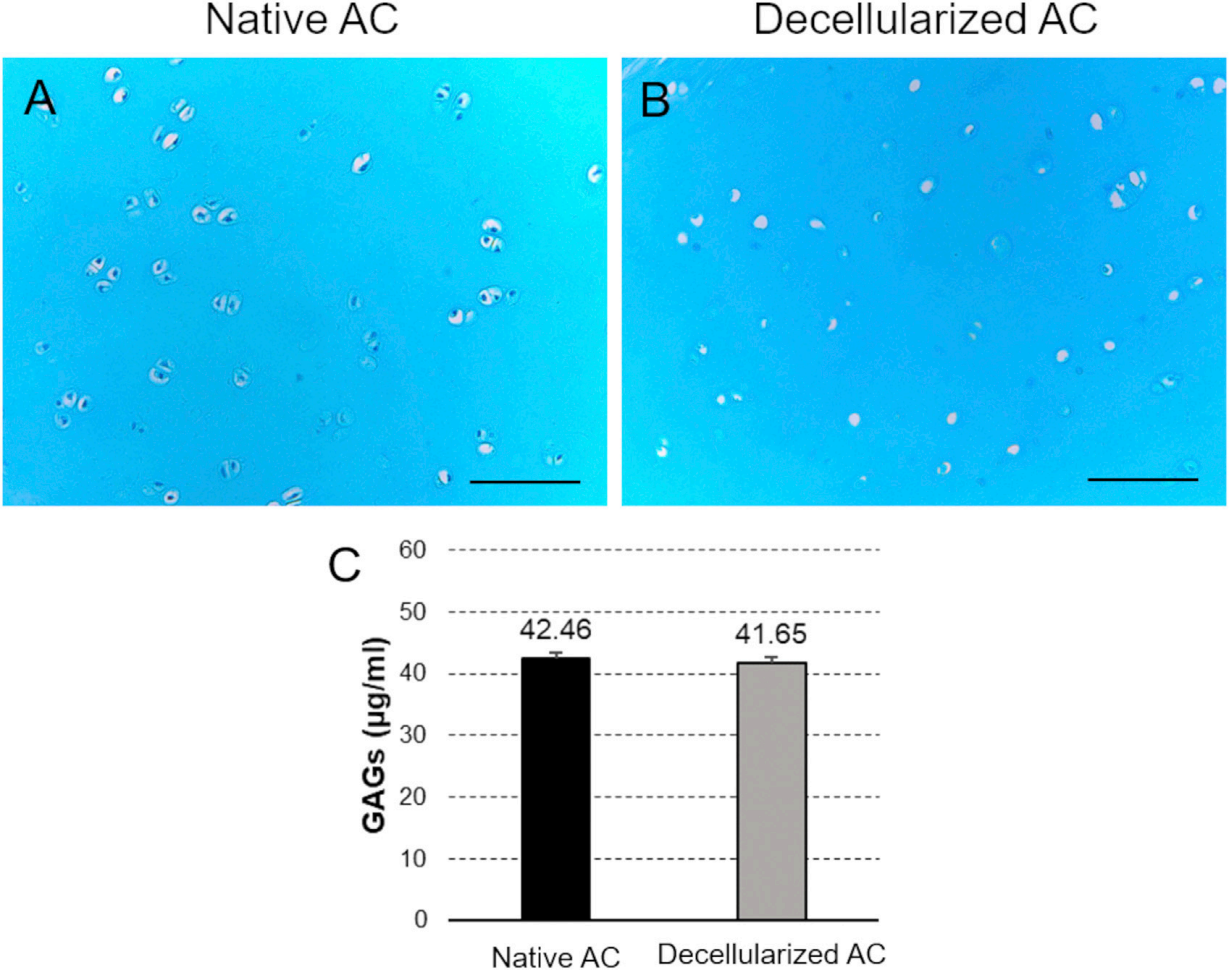
GAG preservation. **(A,B)** Alcian Blue staining of the collagen component into decellularized versus native AC fragments. Scale bar: 100 µm. **(C)** Quantification of GAG content into AC samples before and after the detergent-enzymatic treatment.

The Alcian Blue staining for GAG detection in native and decellularized AC revealed diffuse light blue color of the tissue section in both samples, indicating the preservation of this component into the ground substance of cartilage ECM after treatment (Figures 4A,B). Efficient cell depletion was further confirmed by this staining, showing no detectable cell nuclei in the chondral lacunae of acellular AC. In contrast, light blue-colored nuclei were observed in the native cartilage lacunae (Figures 4A,B).

Additionally, the colorimetric assay highlighted no significant differences in GAG content between decellularized and native AC (Figure 4C).

### 3.2 Scaffold configuration influences surface ultrastructure and porosity

After fabricating the PVA/AC bio-hybrid scaffolds, both macroscopic and ultrastructural SEM analyses were conducted to assess their morphology and structural features.

**FIGURE 5:**
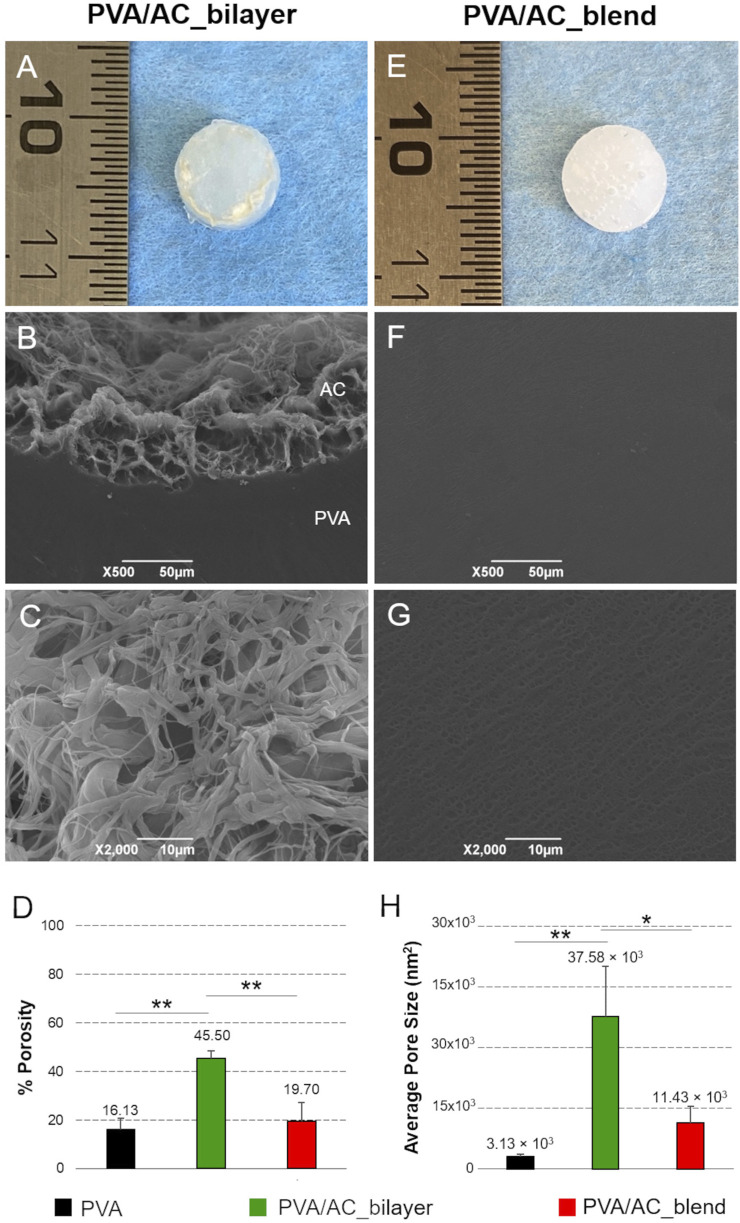
Morphology and ultrastructure of bio-hybrid PVA/AC scaffolds. Gross morphology of PVA/AC_bilayer **(A)** and PVA/AC_blend **(E)** scaffolds obtained after physical cross-linking. SEM micrographs of bio-hybrid scaffold ultrastructure **(B,C,F,G)**. In the PVA/AC_bilayer scaffold, the porous AC layer (AC) is in continuity with the synthetic PVA layer (PVA) **(B)**. At higher magnification, the interconnected porous network of the superficial AC layer is clearly appreciable **(C)**. In the PVA/AC_blend scaffold, nanoscale pores are also present but appear less evident than in the bilayer scaffold because of the lower porosity and smaller average pore size. This results in a more homogeneous surface appearance at the SEM analysis **(F,G)**. Scale bar: 50 µm **(B,F)**; 10 µm **(C,G)**. Morphometric evaluation of scaffold porosity **(D,H)**. (*: p < 0.05; **: p < 0.01).

Macroscopically, the scaffolds displayed a discoidal shape with a diameter of 7 mm and a thickness of 2 mm. In bilayer scaffolds, the presence of a thin layer of decellularized, homogenized, and lyophilized cartilage matrix was clearly identifiable, appearing slightly darker and rougher compared to the underlying hydrogel. On the other hand, in blend scaffolds - where the AC component was mechanically incorporated and mixed with the polymer - the biological material was not visible on the surface and the composites presented a more homogeneous and uniform appearance (Figures 5A,E).

SEM analysis provided further insight into the differences between the two scaffold types. The bilayer scaffold showed a distinct surface profile, with the cartilage matrix layer presenting an irregular, spongy texture. This feature is attributed to the dense network of collagen fibers present in the biological tissue. By contrast, the surface of the blend scaffold appeared more regular and uniform, with nano- and microporosity typical of the PVA polymer (Figures 5B,C,F,G).

Figure 5 is representative of the bilayer and blend scaffolds with both PVA concentrations (15% and 20%), as no difference between the two variants was detected.

The morphometric analysis of SEM images enabled the investigation of scaffold porosity at the superficial surface, which directly interacts with cells during seeding experiments. Semi-quantitative assessments supported the qualitative observations from SEM micrographs, providing further insights into the ultrastructural characteristics of the scaffold surface. Notably, PVA/AC_bilayer scaffolds exhibited a significantly higher porosity (45.50% ± 3.01%) compared to PVA/AC_blend composites (19.70% ± 7.50%, p < 0.01) and control PVA hydrogels (16.13% ± 4.60%, p < 0.01). Moreover, collected data suggest that the mechanical incorporation of acellular ECM into PVA may increase scaffold porosity, even though no significant differences were statistically detected between PVA/AC_blend and PVA samples (Figures 5D,H).

### 3.3 PVA concentration and scaffold configuration influence compressive and consolidation responses

**FIGURE 6:**
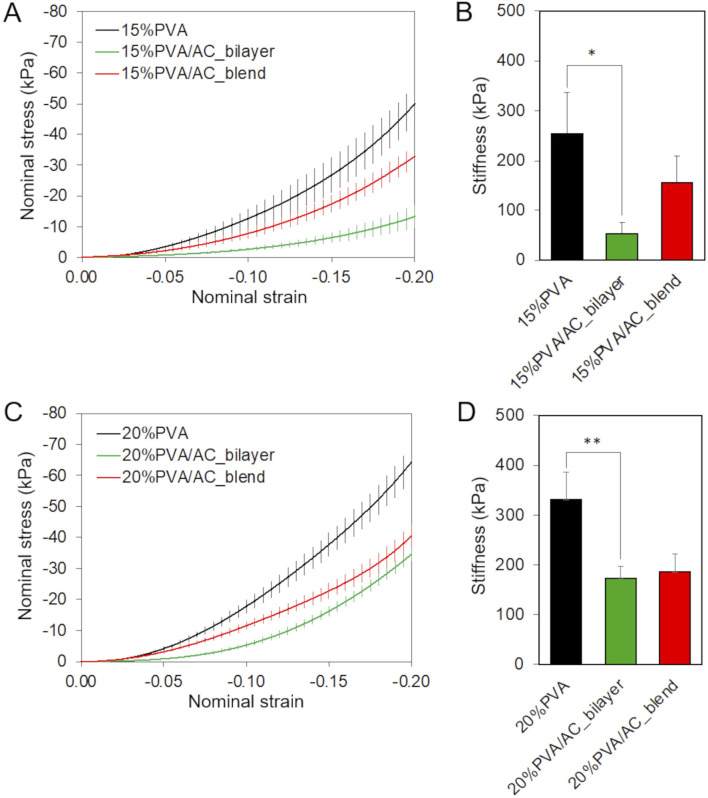
Almost-instantaneous compressive behavior of bio-hybrid PVA/AC scaffolds. Results of compression test carried on 15%PVA **(A,B)** and 20%PVA **(C,D)** scaffolds combined with AC either as a superficial layer or in blend. **(A,C)** Mean + standard error of nominal stress in function of nominal strain. **(B,D)** Compressive stiffness with standard deviation (*p-value <0.05, **p-value <0.01).

The results of instantaneous compressive tests are presented as nominal stress as a function of nominal strain (Figure 6). All the scaffolds exhibited a non-linear compressive behavior. An increment in the compressive stiffness with the hydrogel concentration can be observed. The addition of AC to the scaffolds, whether in bilayer or blend form, led to a reduction in stiffness, with a significant decrease noted in the bilayer configuration (p = 0.017 for 15%PVA and p = 0.004 for 20%PVA).

**FIGURE 7:**
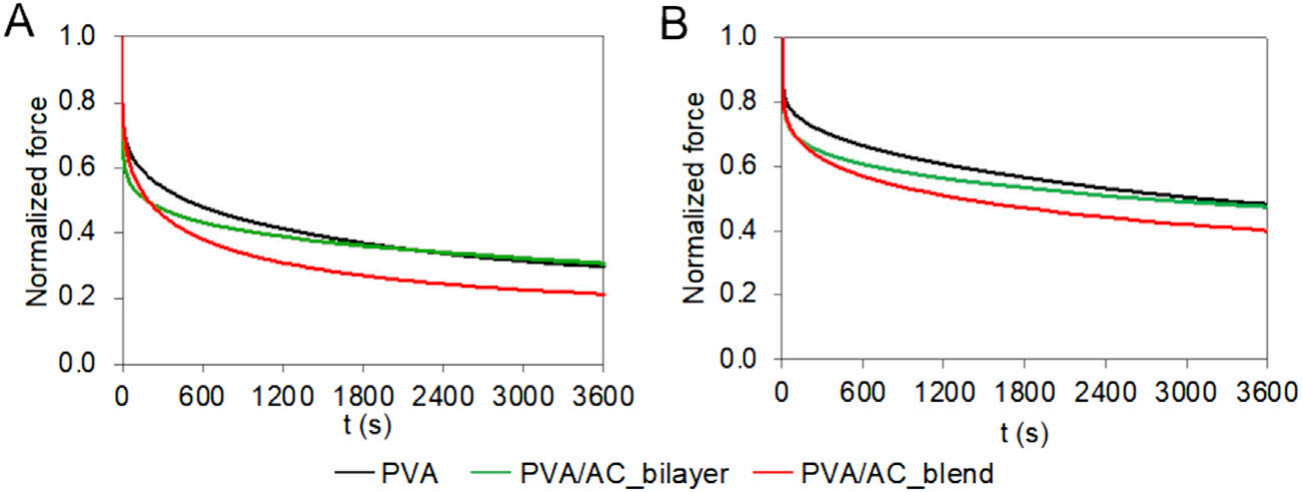
Time-dependent compressive behavior of bio-hybrid PVA/AC scaffolds. Results of consolidation tests performed on PVA scaffolds (15% **(A)** and 20% **(B)**) combined with AC either as a superficial layer or in blend. The curves represent the mean normalized force as a function of time.

The time-dependent behavior of PVA/AC scaffolds was investigated using consolidation tests. The scaffolds based on 20%PVA, the stiffest material, exhibited a smaller reduction in force comparing with 15%PVA. The normalized force decreased by 70% for 15%PVA and 51% for 20%PVA, respectively (Figure 7). For both PVA concentrations, the PVA/AC_blend scaffolds showed a consolidation behavior very similar to the one of non-composite material in the short-term response: indeed, at 10 s both PVA and PVA/AC_blend show a force decrease of about 25%, while PVA/AC_bilayer a decrease of 35%. Instead, at the equilibrium the normalized force reduction was higher for PVA/AC_blend than PVA. Differently, the PVA/AC_bilayer scaffolds showed a larger force drop in the shot time for both PVA concentrations, while at equilibrium the normalized force-time curve overlapped with that of PVA scaffold.

### 3.4 PVA/AC composites support cell adhesion and proliferation

**FIGURE 8:**
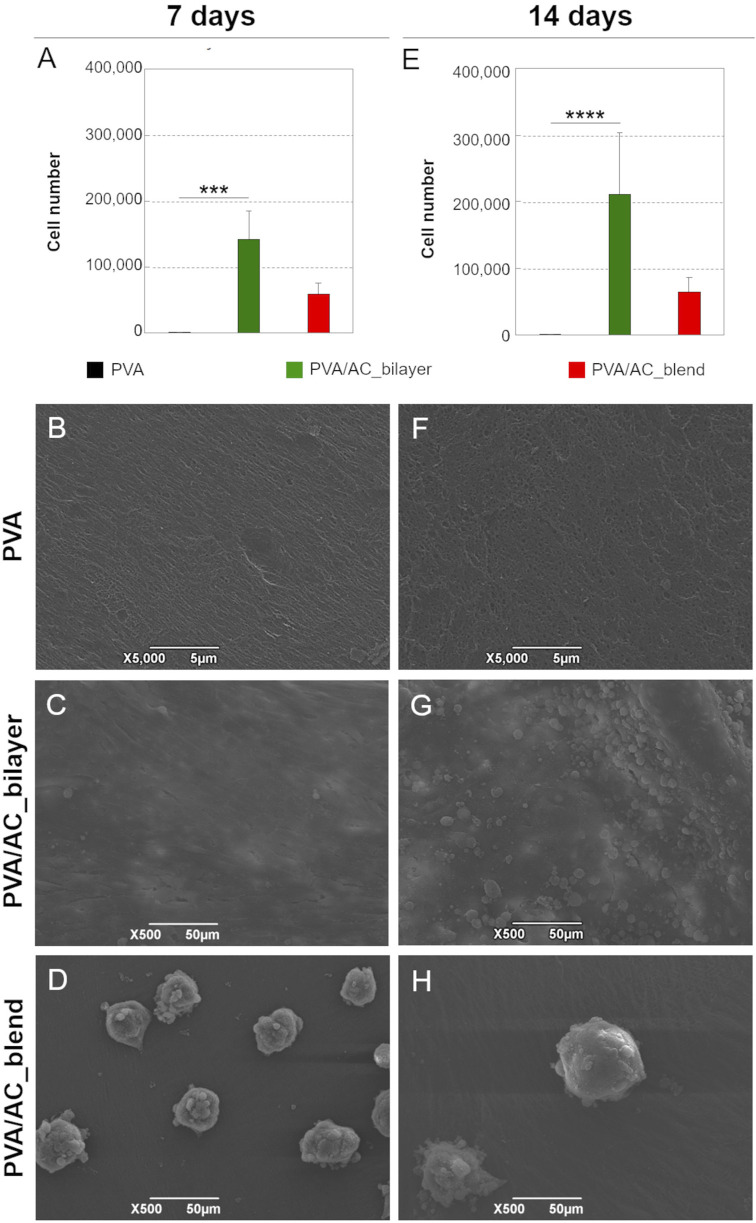
Study of cell-scaffold interactions. **(A,E)** Growth of HM1-SV40 cells on PVA/AC_bilayer, PVA/AC_blend and synthetic PVA scaffolds after 7 and 14 days from seeding (***: p < 0.001; ****: p < 0.0001). SEM micrographs showing the adhesion and proliferation of HM1-SV40 cells on PVA/AC_bilayer **(C,G)** and PVA/AC_blend scaffolds **(D,H)**, compared to PVA scaffolds **(B,F)**. Scale bar: 5 µm **(B,F)**; 50 µm **(C,D,G,H)**.

The cytocompatibility of PVA/AC bio-hybrid scaffolds was evaluated by seeding HM1-SV40 MSCs on their surface and assessing cell viability after 7 and 14 days of *in vitro* culture. For the bilayer scaffolds, cells were seeded specifically on the ECM (decellularized AC) superficial layer, while synthetic PVA scaffolds were used as controls. Cell viability was quantified using the MTT assay, which revealed that, at both time points, the number of cells grown on PVA/AC bilayer scaffolds was significantly higher than that observed on PVA synthetic scaffolds. In contrast, no statistically significant difference in cell growth was observed between PVA/AC_blend scaffolds and pure PVA scaffolds. Moreover, bilayer scaffolds supported a higher number of cells compared to blend scaffolds at both time points. However, this difference was not statistically significant (Figures 8A,E).

The behavior and distribution of HM1-SV40 cells on the scaffolds were analyzed using SEM. As shown in the micrographs in Figures 8C,G PVA/AC_bilayer composites supported the adhesion and proliferation of HM1-SV40 stem cells, which adhered to the scaffold surface, adopting an elongated morphology characteristic of active, proliferating cells. Over time, the cells formed a confluent monolayer on the surface of the scaffold, specifically on the layer composed of decellularized and homogenized cartilage matrix.

Consistent with the MTT assay results, cell proliferation was more pronounced on bilayer scaffolds compared to blend scaffolds. On PVA/AC_blend composites, HM1-SV40 cells displayed a different growth pattern, forming isolated cell aggregates (spheroids) on the surface rather than a uniform monolayer (Figures 8D,H).

As expected, no evidence of cell growth was observed on the PVA-only scaffolds (Figures 8B,F).

## 4 Discussion

Hemophilic arthropathy (HA) remains a significant clinical challenge in the management of patients suffering from hemophilia. In the absence of timely treatment and effective management, this condition can result in severe pain, functional impairment, and potentially irreversible motor disability (Leuci and Dargaud, 2023; Badulescu et al., 2024). Current therapeutic strategies for HA involve coagulation factor replacement therapy to address the underlying deficiency, complemented by orthopedic and physiotherapeutic interventions. However, when these approaches fail to mitigate the ongoing joint damage, surgical options become necessary, with joint replacement being the most extreme solution (Gualtierotti et al., 2022; Rodríguez-Merchán, 2019; Lobet et al., 2014).

Given the challenges associated with partial motor recovery and the elevated perioperative risks, there has been a growing interest in developing non-invasive, regenerative therapies to block the progression of joint damage at its onset. In this context, regenerative medicine and tissue engineering (TE) advocate the use of bio-hybrid scaffolds as a promising strategy for the treatment of cartilage degeneration. (Honarvar et al., 2024; Zhu et al., 2021; Primorac et al., 2024). These novel approaches aim to facilitate the repair and regeneration of AC, specifically targeting focal lesions, and could provide a valuable adjunct to traditional surgical treatments in managing HA.

Starting from these considerations, this work presents a preliminary *in vitro* investigation into the fabrication and bioactivity assessment of bio-hybrid scaffolds composed of PVA and decellularized cartilage matrix, with the perspective of applying them for the repair of focal chondral lesions in patients with HA.

For effective regeneration therapy, the scaffold must replicate both the structural and mechanical properties of the target tissue. Studies on PVA have shown that this hydrogel exhibits viscoelastic properties which may well resemble those of human AC, making it a promising candidate in cartilage TE (Li et al., 2023; Zhong et al., 2024). However, synthetic PVA scaffolds have limited capacity to promote cell adhesion. This was also demonstrated in the present work, where cytocompatibility analyses revealed a lack of cell proliferation on the pure PVA scaffolds, indicating that hydrogel modifications are required to enhance cellular interaction.

To address this primary limitation of PVA and improve its biomimetic properties, research has focused on the development of bio-hybrid scaffolds that integrate the hydrogel with natural materials [revised, for example, by Barbon et al. (2021)]. The incorporation of multiple biological cues that mimic the natural *in vivo* environment is increasingly recognized as essential for supporting complex cellular functions within artificial biomaterials. By integrating biological cues into a synthetic framework, it is possible to create a simplified model that replicates the multifunctional properties of tissue ECM, enhancing the interaction with repopulating cells (Nafea et al., 2015; Di Liddo et al., 2014).

Decellularized ECM is a promising natural material for enhancing the bioactivity of PVA hydrogel. It offers the key advantage of closely replicating the structural and biochemical properties of the target tissue, thereby ensuring high biocompatibility. The decellularization process preserves the native biomolecular composition (i.e., structural proteins, polysaccharides, growth factors) while eliminating immunogenic elements (i.e., cells and DNA), so that the resulting graft becomes immunologically inert. In addition, decellularized ECM is inherently bioactive, that is it can actively influence cell behavior, unlike purely synthetic scaffolds. The retained bioactive elements, such as growth factors/cytokines and structural molecules, provide signaling cues that regulate cell proliferation, differentiation, and migration. This bioactivity is critical for complex tissue regeneration, especially in avascular tissues like cartilage, where natural repair processes are limited (Kafili et al., 2023; Neishabouri et al., 2022).

In this study, a biological matrix derived from human chondral tissue was used to bioactivate the PVA hydrogel, with the goal of developing tissue-specific scaffolds tailored to meet the functional requirements of AC tissue. Combining acellular cartilage with PVA, a polymer known for its mechanical strength and flexibility, bio-hybrid scaffolds can be fabricated with enhanced durability, elasticity, and bioactivity. This makes the system particularly suited for load-bearing applications like cartilage repair.

To the best of our knowledge, we have developed an innovative method for fabricating bio-hybrid PVA-based scaffolds for cartilage TE. Given the limited literature on the bioactivation of PVA with acellular ECM, our work may represent a significant advancement in the field. This innovative approach addresses a critical gap in current TE strategies, offering a promising pathway for the development of scaffolds with enhanced bioactivity and regenerative potential.

Specifically, decellularized AC was processed into a non-structured, homogenized matrix and used in two distinct scaffold designs. In the first approach, the homogenized AC was frozen and lyophilized to form a biological sheet, which was placed on top of a PVA hydrogel, creating a bilayer scaffold with clearly defined biological and synthetic components (Grandi et al., 2018; Stocco et al., 2016; Stocco et al., 2014). In the second approach, the homogenized acellular AC was directly incorporated into the PVA polymer before cross-linking, resulting in a homogenous PVA/AC blend. This dual-fabrication strategy enables the exploration of different scaffold architectures, offering flexibility in tailoring mechanical, structural, and biological properties to better support cartilage regeneration.

Articular cartilage decellularization has emerged as a pivotal technique in TE, aiming to create scaffolds that closely replicate the native ECM for effective structural and functional tissue repair, especially in osteoarthritic patients. Recent studies have focused on optimizing decellularization protocols to achieve efficient cell removal without compromising ECM integrity. Physical (e.g., freeze-thaw cycles), chemical (e.g., the use of chemical detergents) and biological (e.g., enzymatic treatments) methods have been employed, often in combination, to enhance decellularization efficacy [reviewed, for example, by Zhang et al. (2022), Porzionato et al. (2018)]. Despite significant advancements in the field, challenges remain in standardizing decellularization protocols to ensure consistent ECM quality and functionality. Balancing effective cell/DNA depletion with the preservation of essential biochemical cues is critical. Moreover, current studies are also directed toward enhancing the mechanical properties of acellular AC, which are often compromised by the decellularization process. Thus, ongoing research continues to refine AC decellularization techniques, aiming to overcome current limitations and develope simple, cost-effective, and scalable manufacturing processes for clinical translation (Zhang et al., 2022).

In this study, we applied a well-established protocol for human AC decellularization, which combined the use of chemical detergents and enzymatic treatment to successfully balance the removal of immunogenic elements with the preservation of structural biomolecules. After decellularization, the quality of the decellularized cartilage matrix was evaluated through histological and morphometric analyses, which demonstrated that the detergent-enzymatic treatment efficiently removed cell and DNA components of the tissue while maintaining the structural integrity and protein composition (i.e., collagen, glycosaminoglycans) of the ECM in comparison with native AC.

The decellularized and homogenized cartilage was subsequently combined with PVA to fabricate two types of bio-hybrid scaffolds: the PVA/AC_bilayer scaffold and the PVA/AC_blend scaffold. For each kind of composite, both 15% and 20% PVA were tested, but the use of different polymer concentrations did not reveal significant morphological differences, providing similar characteristics in terms of surface ultrastructure and superficial porosity.

The bilayer scaffolds consist of 2 distinct cartilage and PVA layers that remain clearly identifiable under SEM, whereas the blend scaffolds integrate the AC component uniformly within the PVA polymer, with a superficial ultrastructure which resembles the hydrogel. This difference is a key technical factor in the structural and functional properties of PVA/AC composites. The bilayer approach may be advantageous for mimicking the natural architecture of cartilage tissues, where distinct layers with different mechanical and biochemical properties exist. In contrast, the blend method allows for a more homogeneous structure that may improve mechanical integrity but may reduce biological material exposure. Overall, the bilayer scaffolds may offer more targeted cell interaction at the interface of the cartilage layer and hydrogel, potentially improving cell adhesion and differentiation in areas where tissue-specific cues are needed. Related to this, the increase of porosity in PVA/AC_bilayer scaffolds suggests enhanced cell-seeding efficiency and the potential for better tissue integration, especially in applications requiring cellular infiltration. Indeed, scaffold porosity is a key factor in determining the rate of cell infiltration, the formation of tissue-like structures, and the exchange of oxygen and nutrients (Lutzweiler et al., 2020). The larger pore size observed in the bilayer scaffolds likely represents a better support for cell migration and tissue integration, which is essential for cartilage TE. This is consistent with previous findings showing that both the microarchitecture and biochemical composition of acellular scaffolds can drive the interaction between cells and the extracellular environment, modulating their spatial organization and differentiation (Di Liddo et al., 2016). However, excessively large pores could lead to the loss of structural integrity and reduced scaffold mechanical properties (Mukasheva et al., 2024). Furthermore, the effects of pore size on specific cell types, such as chondrocytes or mesenchymal stem cells, should be considered, as these cells have varying size requirements for optimal attachment and proliferation. The porosity of the blend scaffolds, significantly lower than that of bilayer scaffolds and more similar to that of pure PVA hydrogels, still offers sufficient space for cell infiltration and nutrient diffusion. This uniform porosity may contribute to better mechanical properties of PVA/AC composites, which could be advantageous in terms of overall scaffold integrity and load-bearing ability. Nevertheless, the relatively lower porosity could limit deep cell migration compared to bilayer scaffolds, where larger pores in the cartilage layer provide more conducive environments for cell penetration.

These results suggest that PVA/AC scaffold ultrastructure may be tailored according to patient-specific requirements, balancing the porosity with other design parameters to optimize both biological and mechanical performance. In this regard, freeze-thawing (FT) represents a purely physical cross-linking method that enables modulation of PVA scaffold structure and biomechanics through the controlled formation of crystalline domains. It is well established that the number of FT cycles influences scaffold properties, with additional cycles increasing crystallinity and stiffness but reducing swelling capacity and elasticity, while too few cycles may result in mechanically weak and less stable hydrogels (Adelnia et al., 2022; Chen et al., 2021). In the present study, physical cross-linking of PVA solutions was achieved through a FT process adapted from the method originally described by Lozinsky et al. (1995). This procedure was standardized by our group to optimize the structural and mechanical properties of PVA scaffolds, while ensuring reproducibility of features and consistency across experiments (Barbon et al., 2020).

Scaffold ultrastructure is closely correlated with its mechanical behavior, influencing its ability to stimulate cartilage regeneration by providing an optimal support for cell interaction and load-bearing functionality. The mechanical characterization of engineered scaffolds designed for the focal treatment of cartilage damages is crucial because AC must withstand significant mechanical stresses in the body, such as compression, shear, and tensile forces. The scaffold should replicate the mechanical properties of the chondral tissue to provide adequate support during tissue formation, prevent premature degradation or collapse, and ensure proper load distribution to stimulate cellular differentiation and matrix deposition (Ansari et al., 2024; Koh et al., 2019). Accordingly, in this study we investigated the compressive behaviour of PVA/AC composite scaffolds. Compressive tests were performed by applying a deformation of 20% of the total thickness of the samples, which corresponds to the physiological range of maximum compressive strain observed in human cartilage (Zevenbergen et al., 2018; Kabir et al., 2020). The instantaneous compressive tests revealed a non-linear compressive behaviour across all groups, characteristic of this hydrogel (Todros et al., 2022). The hydrogels with highest polymer concentration (20%) exhibited a higher compressive stiffness. This result is consistent with previews studies on other hydrogels (Metters, 2000; Nguyen et al., 2012; Kizmaz and Orakdogen, 2014), indicating that compressive stiffness increases with polymer concentration. Moreover, adding AC to PVA, either in bilayer or blend form, modified the mechanical properties of the hydrogel, due to changes in the overall scaffold structure and alterations of the cross-linked microstructure. Bilayer scaffolds exhibited a more significant reduction in stiffness at maximum compression: indeed, at the maximum deformation level considered, the AC layer was almost exclusively compressed, which is the less stiff layer, while PVA layer was not much affected by compression. Erickson and collaborators (Erickson et al., 2019) observed a similar behaviour in bilayer scaffolds composed by chitosan-hyaluronic acid and chitosan-alginate enriched with hydroxyapatite nanorods. In their study, two linear-elastic regions (first region–low stiffness: from 0% to 30% of strain, second region–high stiffness: from 40% to 70% of strain) were identified, suggesting that the low stiffness region is compressed prior to the stiffer region. The compressive stiffness values measured for all scaffolds, except for the 15%PVA/AC, fell within the range of compressive stiffness for human articular cartilage, approximately 200 kPa–5400 kPa (Belluzzi et al., 2023).

When subjected to compressive loads, cartilage, characterized by a high water content, undergoes consolidation. This phenomenon consists in fluid exudation and compaction of the solid phase and results in friction reduction due to hydraulic lubrication and attenuation of sudden loads (Biot, 1941; Broom and Oloyede, 1998). During consolidation tests on both native and composite PVA/AC scaffolds, we observed that, in the short-term, the PVA behaviour was followed by the blend scaffolds, while at equilibrium, by the bilayer. This suggests that for the bilayer scaffold, initially the compressive loads are sustained by the superficial layer of AC, while at equilibrium the underlying PVA bulk supports the loads. In contrast, the blend scaffolds, instantaneously respond like the non-composite material, but over time, both the PVA and AC rearrange to sustain the loads, reducing the compressive force. Finally, the force reduction observed for PVA/AC_blend (79% for 15%PVA/AC_blend, 60% for 20%PVA/AC_blend) is comparable to the values reported for articular cartilage under compressive conditions (June et al., 2009).

The final step in the *in vitro* validation of PVA/AC bio-hybrid scaffolds involved conducting cell seeding experiments. Cytocompatibility and bioactivity studies are essential for evaluating the regenerative potential of engineered tissue substitutes, ensuring that the scaffold materials are non-toxic to cells and provide a conducive environment for cellular growth. PVA hydrogels in the 10%–30% range, including the 15% and 20% formulations used in this study, are well documented to be non-toxic and biocompatible, although their hydrophilic nature prevents cell adhesion (Stocco et al., 2014; Liang et al., 2024). In the bio-hybrid scaffolds, the cell-interactive properties derive from the coupled AC-derived matrix, making the biological response representative of both PVA concentrations within the composites. For this reason, and to optimize sample use, biocompatibility assays were performed on the 20%PVA-based supports, while the choice of polymer concentration can be tailored to the mechanical requirements of the implant site. Under these conditions, seeding experiments with the HM1-SV40 cell line confirmed the cytocompatibility and bioactivity of the PVA/AC scaffolds, specifically their ability to support cell adhesion and proliferation. The MTT assay revealed enhanced cell proliferation on the bio-hybrid scaffolds in comparison to PVA control samples, with better results obtained for PVA/AC_bilayer supports. This indicates that that cell growth was influenced by the bio-active properties of the cartilage matrix, which was more effective when it was kept distinct from the synthetic polymer. When seeded bio-hybrid scaffolds were analysed by SEM, distinct cellular behaviours were observed on the two types of samples. On PVA/AC_bilayer scaffolds, cells adhered and proliferated as a two-dimensional (2D) monolayer, colonizing the surface consisting of the cartilage matrix. In contrast, on PVA/AC_blend scaffolds, cells formed three-dimensional (3D) self-assembling cellular aggregates called spheroids.

Within the field of tissue engineering (TE), 3D cellular constructs offer significant advantages over conventional 2D cell cultures. Unlike monolayer cultures, 3D constructs more accurately mimic the physiological activities of biological tissues, including cellular organization, cell-cell interactions, and cell-extracellular matrix (ECM) interactions (Chae et al., 2021; Duval et al., 2017). The formation of spheroids is typically favoured when cells are seeded on scaffolds with limited adhesive properties, suggesting that the PVA/AC_blend supports have a lower capacity for promoting direct cell adhesion than the bilayer counterparts. Nevertheless, the enhanced bioactivity of the blend scaffolds, compared to pure PVA scaffolds (on which no cell proliferation was observed, either as a monolayer or in 3D), was highlighted by their ability to support the formation of 3D cellular aggregates. These spheroids, due to their microstructural organization, better replicate cellular functions - such as viability, differentiation, and paracrine signaling - compared to traditional 2D cultures (Habanjar et al., 2021).

As future perspective, cell studies should be integrated with differentiation experiments to evaluate scaffold ability to guide stem cells toward a chondrogenic lineage. These experiments will offer valuable insights into the scaffold capacity to replicate the biochemical and biomechanical signals of native cartilage ECM, which are crucial for stimulating the synthesis of matrix-specific markers such as collagen type II and GAGs. Notably, this research represents an essential step before *in vivo* testing, as it allows to test the scaffold potential to support the formation of functional cartilage tissue and ensuring its translational viability for clinical applications.

Overall, this study demonstrates the promising ultrastructural, mechanical, and functional properties of PVA/AC bio-hybrid scaffolds, supporting their potential application in focal articular cartilage repair, including in patients with HA. From a translational perspective, PVA/AC scaffolds present important advantages. PVA provides mechanical strength, elasticity, and stability under load, properties that are crucial for mimicking the native joint environment. The incorporation of AC-derived ECM introduces essential bioactive cues, enabling cell adhesion, proliferation, and matrix deposition, thus overcoming the intrinsic bio-inertness of PVA. The bilayer and blend configurations further allow the modulation of cell behavior, offering flexibility depending on the therapeutic goal. At the same time, some limitations remain. PVA is non-degradable *in vivo*, potentially restricting long-term remodeling, and AC sourcing requires cadaveric donors with standardized decellularization, which increases variability and production complexity. Cost-effectiveness and scalability must also be evaluated in comparison with fully synthetic or recombinant alternatives. Finally, the absence of *in vivo* testing leaves open questions on integration, host response, and durability in the hemophilic joint microenvironment.

## 5 Conclusion

The present study developed and evaluated PVA/AC bio-hybrid scaffolds in bilayer and blend configurations using 15% and 20% PVA, with the aim of supporting articular cartilage regeneration in HA. The scaffolds displayed structural integrity and mechanical properties approaching those of native cartilage, with the 20% PVA variant providing superior stiffness. Importantly, the incorporation of AC endowed the constructs with biological functionality: bilayer scaffolds promoted 2D cell adhesion and proliferation, while blend scaffolds facilitated 3D spheroid formation, confirming the essential bioactivity of the ECM fraction absent in pure PVA controls.

Overall, this work represents a significant advancement in the design of bio-hybrid scaffolds for AC repair, offering a system that combines the mechanical properties of PVA with the biological cues of decellularized cartilage ECM. To further advance this approach toward clinical translation, future studies should focus on stem cell-based assays, dynamic mechanical stimulation, and *in vivo* validation into animal models of cartilage damage. However, this scaffold design strategy holds promise for developing next-generation implants that address the limitations of current cartilage repair methods, in the perspective of offering a more effective, scalable, and patient-specific solution for HA management.

## Data Availability

The raw data supporting the conclusions of this article will be made available by the authors, without undue reservation.
